# A Practical Treatise on Diabetes

**Published:** 1830-07-01

**Authors:** 


**A Practical Treatise on Diabetes: with Observations on the Tabes Diuretica, or Urinary Consumption, especially as it occurs in Children; and on Urinary Fluxes in general. With an Appendix of Dissections and Cases, illustrative of a successful Mode of Treatment: and a Postcript of Practical Directions for examining the Urine in these Diseases. By *Robert Venables*, M. B. Physician to the Henley Dispensary, &c. &c. Octavo, pp. 214.**

We must accord to Dr. Venables the credit of great zeal in the prosecution of his profession, and possession of a very respectable quantum of talent for the attainment of his laudable objects. Attached to a public institution, he loses no opportunity of acquiring that knowledge, therapeutical and pathological, which cannot easily be got, except through the instrumentality of such asyla for the indigent portions of society—and what is still more praiseworthy, he freely communicates the results of his observations and researches to his brethren and the public at large. Such conduct deserves every encouragement, however defective may be the attempts of individuals to add to the common stock of our knowledge; and the critic, who thoughtlessly deals out his strictures upon such undertakings, does not contribute to the public good, however he may plume himself on the superiority of his own penetration and acumen. It is much easier to criticise a book than write one, and the medical as well as the general reviewer should sometimes bear in mind the words of the great English satirist, when he dips his pen in gall:—

> Let those judge others who themselves excell,
> And censure freely who have written well.

Dr. Venables has undertaken a practical rather than a literary history of diabetes; and if any one is disposed to ask, has he any thing new to introduce, to compensate for the trouble of reading his book, this is his answer—"I have presented him with two facts in the history of diabetes, which are certainly worthy of attention: first, that an excessive discharge of urine is frequently a cause of tabes in children: secondly, that phosphate of iron proves, when properly administered, almost as certain an astringent upon the excessive action of the kidneys, as opium upon that of the alimentary canal." These are facts which Dr. Venables avers that he has ascertained, and he has appended the history of several cases, in order that the reader may judge for himself.

Our author differs from some respectable authorities on the pathology of diabetes. He thinks we have no occasion to look to the sugar of vegetables separated in the stomach, to account for the saccharine properties of diabetic urine. "The morbid action of the kidneys is quite sufficient to account for the fact, and it is certainly infinitely more rational, and far more consistent with the doctrines of physiology, to attribute the evolution of sugar in the urine to a wrong or perverted action of the glands." Dr. V. has not attempted any division or subdivision of diabetes into the insipid, mellitic, serous, &c. The diabetes of the adult and of the child, as far as he is acquainted, arises from the same causes, and is to be treated in the same manner—hence there are no just grounds for division of the subject.

"There is a cause of emaciation among children, which has hitherto attracted but little of the attention of the Profession. I have often observed children to all appearance very healthy up to a certain period, when suddenly the constitution changes, the child emaciates, its health declines, and, without any obvious derangement sufficient to account for the gradual depravation of health, at last dies a most miserable object. In such cases, the head, chest, and abdomen, present no morbid appearances sufficient to account for the wasting and gradual decline of health. Accident led me to a discovery of the real seat of disease in such cases; and when the history of the complaint has been submitted to the reader, he will not be surprised that its nature and seat should have so long escaped general observation.

"Several cases of wasting having presented themselves to me, in which I was unable to detect any serious or permanent derangement of function—vital or animal,—I was at a considerable loss to account for the phenomena, as well as to direct the treatment. Occasionally the bowels were out of order, but their healthy functions were readily restored, without, however, any sensible effect upon the progress or severity of the disease. Sometimes dyspnœa attended, but evidently of a nervous nature; for the means which were applicable to the primary or secondary diseases of the head, speedily subdued this symptom, when those directly applicable to pulmonary affections proved, when not injurious, wholly useless. The head often seems to be the seat of disease, if we were to judge from the dull comatose state of the patient; but yet, upon dissection, the brain in many instances presented no diseased appearance whatever. In other cases, a partial but slight degree of vascularity has been observable, by no means, however, sufficient to account for the gradual, but commonly fatal, progress of the disease. From the tumid prominent abdomen, which generally, though not universally, prevails under these circumstances, we are naturally led to anticipate traces of disease in the alimentary tube, or some of the digestive organs. But dissection proves that our speculations are unfounded, for with the exception of flatus, which would of course account for the abdominal swelling, I have not been able to discover any other morbid appearances in these organs. When flatus has not been discovered, some slight thickening of the coats of the intestines may be observed, but in no way sufficient to account for the general symptoms." 5.

Accident first led Dr. Venables to discover the real nature of the case. Upon one occasion he was given to understand that the different functions of the child were regularly and healthily performed. The head was free from pain, the respiration natural, the bowels free, and the secretions from them healthy. He was told there was nothing remarkable in the urine, but he found that it was discharged in very great abundance. This was not noticed, as it was attributed to the inordinate quantity of fluid taken in to satisfy the thirst, with which the little sufferer was harrassed. When our author had ascertained that the wasting of the body, in this case, was attended by an excessive discharge of urine, he had little difficulty in viewing them as cause and effect. On more minute examination he discovered diseased appearances in the kidneys sufficient to account for all the symptoms. He ventures to assert that many children have been treated for hydrocephalus, mesenteric affection, rickets, &c. who have really fallen victims to this form of disease. The urine of children is so little attended to, either by mother, nurse, or medical practitioner, that derangements of this secretion too often escape detection. Diabetes indeed is seldom observed in its incipient stage; and when it has made progress, it simulates so many other diseases, that the real character of the complaint is not developed till its history is either wholly lost, or so confounded with symptomatic or secondary affections, that it can no longer be unravelled. Our author next proceeds to a graphic description of the disease, more especially as it affects children; and this being among the most original portions of the volume, our quotation shall be more free than usual.

"The disease seldom if ever appears till after the child has been weaned. The reason of this, perhaps, is, that the exciting causes are seldom applied till after this period. A child which has continued healthy up to this time, will perhaps suddenly lose its usual flow of spirits, become dull and inactive, and although no obvious disease may be recognisable, yet the child will not appear in its usual health. It begins after a very little time to waste in flesh, and then gradually continues to emaciate. The skin becomes harsh, dry, and flabby, and seems to hang loosely about the body. The temperature is generally very much elevated, and, in the description of nurses, it will be said that 'they burn like a coal of fire.'

"In the early stages of the disease, the bowels are regular, and little or no deviation from the natural and healthy appearance of the alvine discharges is remarkable. The tongue also at the beginning indicates no symptom of disease, but when it has continued for some time, and produced some degree of fever, then the tongue becomes covered with a coat of mucus. After a continuance, the bowels begin to act irregularly, the appearance of the evacuations deviating from that of health. Sometimes they are of a greenish hue, at other times they appear natural when passed, but become greenish some time after being voided. In adult cases, constipation very generally attends.

"At a more advanced period, the abdomen seems preternaturally full and distended. The abdominal prominence frequently leads to the supposition of mesenteric disease, an opinion which is still farther confirmed by the progress of emaciation. I doubt much if the mercurial purges, which are exhibited under such circumstances, be wholly innocent. I have some reason to suspect that they have done considerable harm.

"The pulse from the first is generally accelerated, and has a hard, wiry feel. Those who are much in the habit of examining the pulse in children, would recognise in the sensation which the pulse at this time gives, an indication of very great irritation in the vascular system.

"The most remarkable symptom, however, although it frequently escapes observation, is the inordinate discharge of urine. This discharge increases in quantity so gradually, that it is not usually noticed. By the time it has become more remarkable, great thirst prevails, and hence it is neglected or unnoticed, because the parents and friends conceive an excessive discharge of urine, and an excessive consumption of fluid, as naturally associated.

"With respect to the qualities of the urine, they will be found to vary in different cases. In some, the urine appears quite limpid; in others, it appears milky, or like a mixture of chalk and water. Sometimes it is of a pale straw colour; and in a case which is at this moment under my care, I find it is of a green colour. The urine sometimes seems milky, dense, and its specific gravity is much increased. It frequently coagulates by heat, or by the addition of the different reagents. When the quantity of coagulable matter bears any thing like an equal ratio to the watery portion of the urine, and the discharge is much increased, the emaciation under such circumstances proceeds rapidly and extensively.

"As the duration of the disease is prolonged, other symptoms, proportioned in some degree to its severity, set in. Frequently the sensorium becomes affected at an advanced stage; hence headach, vertigo, and even temporary delirium, occasionally attend. When a fatal termination takes place, the patient often dies comatose, and sometimes apoplectic.

"The skin is usually dry and harsh to the touch, and this whether there be fever or not. Generally, however, at an advanced period, there is a considerable degree of fever. As the disease advances, the patient is attacked with remittent fever, occasionally accompanied with profuse perspiration at night. This fever has been regarded as partaking of the character of hectic; and should any cough, with or without expectoration, be present, (by no means an unfrequent occurrence,) the patient may be considered as labouring under phthisis, instances of which I have occasionally seen.

"When diabetes has continued for a long period, it frequently terminates in anasarca or general dropsy. Hence the ancles become œdematous, and the patient, from having been reduced to nearly a skeleton by emaciation, becomes bloated from the accumulation of dropsical fluid in the cellular membrane.

"In children, about this period, the abdomen becomes sensibly enlarged. This enlargement, by a careless observer, may be mistaken for ascites, or some mesenteric affection. Ascites frequently supervenes in adults, but more rarely in children. Disease of the mesentery is to be regarded as an adventitious rather than an essential occurrence." 16.

In diabetes the digestive function is, of all others, the most liable to disorder. Dr. Venables thinks it the consequence rather than the cause of the diabetes.

*Morbid Anatomy.*

This our author confines to the kidneys and urinary organs. He avers that "diabetes never exists to any extent, without the kidneys presenting on dissection manifest changes."

"These changes vary from a trifling vascularity to severe organic derangement. Sometimes the kidneys are much inflamed, and present a florid vascular appearance, in other cases the venous system of these organs seems enlarged and turgid with blood. In a case which I examined about five years ago, the kidneys were enlarged in size, dark-coloured, and seemed turgid with blood. On cutting into the substance, there was an instantaneous effusion of fluid dark-coloured blood, as happens when a congested liver is cut into. Sometimes the veins, on their external surface, form a complete net-work of vessels. In some cases the kidneys are found in a loose, flabby state, being at the same time much increased beyond their ordinary size. They are often of a pale or ash-colour.

"In some instances the substance of the kidneys is much inflamed, and then they present an appearance of a high degree of arterial vascularity. Their substance feels dense, and their structure firm. Frequently, under such circumstances, a whitish fluid resembling pus is found secreted in some quantity in the infundibula.

"The kidneys do not often contain abscesses, but I have seen two cases in which they were ulcerated. In these cases, the pus occasionally passed with the urine, and was mistaken for flakes of coagulable lymph, which it very much resembled. The ureters are often enlarged in diameter; and a respected medical friend informed me, that he once saw a case in which the internal surface of one ureter was ulcerated. It is natural enough to expect that these vessels should be enlarged in such a disease; but I have not met with a case of ulceration. This, however, may have been owing to my not having been prepared to expect, and, consequently, not having uniformly looked for such an effect. The renal or emulgent arteries are very often found larger in diameter than natural. Generally speaking, both kidneys are diseased, but sometimes only one, or at least only one to any great extent.

"The bladder is sometimes found rather vascular, and turgid on its mucous surface; sometimes the mucous surface is inflamed. The substance of this organ, is, in some cases thickened, and very firm in its texture. I saw a case, which was examined by an eminent surgeon* in Dublin, about fifteen or sixteen years ago, in which the mucous coat of the bladder was tuberculated, and elevated into large, thickened, hard, and irregular plicæ. In several spots it was exulcerated, and, in this case, I learned that there was frequently a considerable discharge of sanio-purulent urine.

"There are diseased appearances occasionally observed in the other viscera, as the brain, lungs, heart, liver, spleen, pancreas, and the other digestive organs; but, as a great variety of these occur, and as diabetes frequently prevails without as well as with them, and sometimes with one description, and sometimes with another,—they are to be regarded rather as accidental occurrences, than as absolutely and essentially a part of the morbid anatomy of the disease; and therefore their consideration can have no place here." 20.

*Remote Causes.*

Among these our author ranks all those agents which inordinately stimulate the kidneys, as spirituous liquors, excesses in acids, alkalies, strong diuretics, mercurial courses, eruptive diseases, particular articles of diet, as asparagus, foreign acescent wines, intemperance of every kind, inordinate bodily exertions, blows upon the loins and region of the kidneys, strong mental emotions, hereditary disposition depending on peculiar structure of the kidneys, derangement of the digestive organs, though not so frequently as Dr. Rollo would wish us to believe.

*Immediate Causes.* Dissection, according to our author, has shewn that this disease is invariably attended with some manifest change in the *structure* of the kidneys, and hence he thinks that a mere functional disorder is hardly adequate to the production of the permanent affection. Of the precise nature of these organic changes we are at present ignorant.

*Pathology.* By this term our author means "those morbid operations by which morbid effects are produced"—in other words, the mode in which the remote or exciting causes act in producing diseases. Dr. Venables makes several weighty objections to Dr. Rollo's doctrine of diabetes.

"Were Dr. Rollo's views not otherwise objectionable, the fact that the urine, in many instances, is not saccharine, until after some continuance of the disease, would be alone sufficient to invalidate them. If the saccharine properties of the urine were owing to the separation of this substance by the kidneys, from its commixture with the blood through the faulty action of the stomach, it naturally follows that the urine, from the first augmentation of its quantity, should contain saccharine matter. Here, then, are two strong objections to Dr. Rollo's theory; first, the blood does not in every instance contain sugar; secondly, the urine is not saccharine until the disease has lasted for some considerable time." 32.

After some experiments which our author made on the state of the urine upon the ingurgitation of certain fluids, he comes to the conclusion "that diabetes more frequently arises from a peculiar excitement of the kidneys originating in the direct application of stimuli to their substance."

* Mr. Peter Harkan.

"At first the excitement is only occasional, and the effect subsides; but the repeated irritation of organs, we well know, brings on inflammatory action, and at last disorganisation. It has been observed, in discussing the morbid anatomy of the kidneys in diabetes, that they generally exhibit morbid vascular appearances, and frequently considerable disorganisation of their structure. The first effect of the irritation is merely an increase of their natural functions, and more urine is separated, than under their natural action would be effected. The qualities of the urine, too, are not affected, or at least not sensibly so, at first, nor until after the repeated application of the stimuli. But, by repeated excitement, not only are their functions increased and sensibly perverted, as is indicated by the coagulation of the urine, and its saccharine properties, but also their structure and organization become seriously affected." 38.

We shall pass over a great deal of this chapter, and also that on *diagnosis*, since few people can mistake the disease, if they make a proper investigation of the symptoms. We have seen these, however, overlooked by men of great capacity, till a patient was at the verge of the grave, and that from mere inattention. Emaciation, thirst, voracious appetite, dry harsh skin, with a frequent and copious discharge of urine, especially if it be saccharine, are sufficiently characteristic of this terrible malady.

The *Prognosis* in diabetes has been generally unfavourable. Dr. Venables is not quite so desponding, especially where the disease is early detected.

"We may generally infer, (*cæteris paribus*,) the earlier medical treatment has been instituted, the greater the probability of a perfect recovery. When, from the duration of the disease, we have reason to suspect serious organic changes in the structure and mechanism of the kidneys, we must then be more guarded in our prognosis, and not excite hopes or expectations which probably will never be realised." 48.

Our author has seen recoveries effected "under the most unpromising circumstances," but these are no more than exceptions to the general rule. Our prognosis, in fact, must be founded on the state of the constitution as a whole. Where it is bad there is little hope—where no other particular organ or function is threatened than the kidneys and their secretion, some chance is left.

### Treatment.

The first object, in our author's therapœia, is to restrain the inordinate action of the kidneys, and therefore it will be necessary to search for the real exciting causes, and remove them, if possible. No treatment can be successful while the causes that produced them continue to act. If an infant, the nurse's milk should be examined, and if found to be acescent, the nurse should be changed. If an adult, a strict inquiry should be made into the diet, habits of life, &c. The mere removal of causes or correction of bad habits, however, will not cure the disease, if established. There is one fact, which we have learnt from experience, namely, that excessive discharges from the system are moderated by bleeding, independent of any inflammatory condition obtaining at the time; and in this way, Dr. V. observes, venesection may have often proved a powerful means of restraining the urinary flux, in cases where there was no indication of inflammatory action in the kidneys. But as dissection has often shewn a turgid vascularity of those organs, the utility of venesection becomes still more evident. Dr. Venables' experience coincides with that of Dr. Watt of Glasgow, and some other writers, on this point of practice in diabetes; and he almost invariably adopts venesection, either as a preparatory or curative measure.

"We should not be deterred from repeating the bleedings, merely because the blood does not exhibit the buffy coat, the usually received characteristic of inflamed blood. I have in another place suggested the probability of the characters of the serum being capable of indicating an inflammatory state of the system. I have generally found that a dense milky appearance of this part of the blood indicates inflammatory action, and this independently of the appearances presented by the coagulable part. I have found the pulse rise under such circumstances after venesection, and a repetition of the operation required; although the crassamentum should not exhibit the buffy coat, but even seem infirm and dissolved." 59.

The extent to which the measure should be carried can only be judged of by the practitioner in attendance. Repeated small bleedings, however, are preferred by our author to fewer large ones. General bleeding is the best; but if in infants, leeches to the region of the kidneys may be a good substitute. Blisters to the neighbourhood of the plethoric organ are advised by Dr. V. but caution is necessary lest they stimulate the kidneys. They should be kept open by the ceret. sabine rather than the lytta. If there is reason to suppose that considerable organic disease obtains in the kidneys or spinal marrow (a part suspected by our author) then caustic issues should be employed.

Our author makes many judicious observations on the various internal medicines which have been recommended in diabetes. He thinks they have acted more by increasing other secretions than by restraining directly the urinary discharge. After speaking of various tonics and restringents, our author proceeds thus:—

"These facts led me to the conclusion, that some of the metallic phosphates might be advantageously substituted for those with an alkaline base. The tonic and astringent properties of iron and zinc pointed them out as the best suited to the object in view. I selected iron for my first trial, and I have felt so satisfied with its powers, that I have not attempted any farther investigation. I have been really struck with the efficacy of the phosphate of iron in excessive discharges* of urine. The quantity is rapidly reduced under the use of this salt, and indeed its qualities sensibly altered. The bulimia which also attends on diabetes is reduced, and the powers of digestion invigorated and increased.

"The phosphate of iron is readily formed by the admixture of solutions of sulphate of iron and phosphate of soda. The resulting salts are sulphate of soda, which, being soluble, passes through, while the insoluble phosphate of iron remains on the filter.

"Phosphate of iron may be given as an astringent in doses of one or two grains, which may be gradually increased to a scruple or half a drachm three or four times in the day. In children, smaller doses should be given, but the

* "In rickets, carbonate of iron is usually combined with the phosphate of lime, and the combination is found more efficacious than either singly. I have no doubt that decomposition takes place, for in the animal laboratory, the laws of chemical affinity are set at defiance, and those compounds evolved which are most suited to the living purposes."

exposition of the rules for apportioning them according to the ages of patients, belong to a different branch of medicine. It may be observed, that after a continued use of any medicine the dose must be gradually increased, or otherwise its effects will begin to diminish. Sometimes it is useful to suspend the use of the medicine for a short time, and then to recommence it again. In this way the susceptibility of the system is often revived, when it would not be safe to attempt the same object by any other means." 72.

Of the various disorders which accompany diabetes, and which are looked upon by some as the causes of the disease, Dr. Venables has given a full account, with very clear and judicious directions for the treatment of them. We refer the reader to the seventh chapter for very excellent therapeutical and dietetic observations. The eighth chapter also, on Prophylaxis, is worthy of attentive perusal.

In an extensive appendix, our author has detailed eighteen cases, and two dissections of diabetes. Of these, ten are examples of the urinary flux of children, and form the most novel, and, we think, the most important part of the work. On this account we shall endeavour to convey to our readers a sketch of some of the cases to which we allude.

*Case* 1. A little boy, aged five years, had been ailing for nearly two years. He first became dull and listless, and then emaciated. He frequently complained of his head—eyes were prominent—pupils rather dilated—bowels occasionally disordered. In the progress of the complaint the abdomen swelled a little, and mesenteric affection was suspected. The appetite was always good. Cough took place, and then phthisis was feared. Several practitioners were consulted, and various plans of treatment were adopted, without benefit. The child ultimately came under Dr. Venables. He also tried various means, abandoning one after the other. The little patient at length became generally dropsical and died. It was now ascertained that, previously to the dropsical symptoms, the boy had had a great flow of urine—sometimes as much as eight pints per diem. When the dropsy supervened, the urinary secretion diminished.

*Dissection.* There was nothing remarkable in any of the abdominal viscera, except the kidneys. These organs were found in a very diseased state. The right kidney was enlarged, and its vessels turgid with blood. The substance of the viscus felt soft and flabby to the touch, and on cutting into it, a dark-coloured fluid flowed out in abundance. The left kidney presented nearly a similar appearance, but not to so great an extent.

*Case* 2. A woman, who had suffered from diabetes for a long time, at length became dropsical and died. She had always complained of severe pain in the loins. On dissection, the spinal marrow was examined, and the theca vertebralis was found very vascular—the medulla spinalis itself felt unusually hard throughout its whole extent. In one spot it seemed dissolved or softened down into a kind of excavation, not apparently from any thing like ulceration, but as if it had been scooped out at that spot. The excavation was about half an inch in length, situated between the last dorsal and first lumbar vertebræ. A degree of low inflammation seemed to have pervaded a considerable portion of the column, both above and below the excavated part, as evinced by depositions of coagulable lymph, &c.

This case has been introduced with the view of exciting the attention of practitioners to the examination of the spinal column in diabetic dissections, in order to ascertain whether that organ bears any part in the pathology of the disease. We are of opinion that, in the present case, the two affections were mere coincidences. This, however, is only matter of opinion.

*Case* 3. Our author accidentally saw a little child about eighteen months old, that had been weaned ten months. After weaning its spirits failed, its health declined, and it gradually wasted, till it appeared like a skeleton. The appetite was, all this time, voracious, and the thirst insatiable. The abdomen was full, prominent, and hard to the touch. The bowels alternately costive and relaxed—stools frequently green—skin hot and dry, but loose and flabby—cough. It was said that the urine was natural, but, on particular enquiry, it was found to amount to four pints in twelve hours. It had a wheyish appearance, but little taste or smell—did not coagulate by heat or nitric acid. After opening the bowels, chlorine was administered, and then two grains of the phosphate of iron, half a grain of rhubarb, and three of the pulvis aromat. thrice a day. In a fortnight or three weeks the urine was reduced to a quart in the twelve hours. The phosphate was gradually increased, and in six weeks the urine was reduced to the natural quantity. The appetite had now entirely failed—the phosphate was left off, and proper means were used to restore the general health. The child perfectly recovered.

*Case* 4. A boy, aged ten years, had been declining for upwards of two years. There was now considerable emaciation, with head-ache, thirst, occasional nausea—pulse hard, frequent, and contracted—cough—dyspnœa—hurried respiration—abdomen full and prominent—bowels alternately constipated and relaxed—evacuations of a good colour—skin dry—febrile heat—appetite keen—urine eight pints per diem, containing a good deal of coagulable lymph, and was sensibly saccharine. The boy had dull obtuse pain in the back and region of the kidneys. Hydrocephalus had been suspected, and mercury employed, but no relief was obtained from any treatment. Dr. V. ordered blood to be taken from the arm, and small doses of antimonial and Dover's powder to be given, at proper intervals—the bowels to be kept open by senna and jalap. The blood being buffed and cupped, venesection was repeated, at first weekly, and afterwards monthly, gradually diminishing the quantity. Leeches were applied to the loins, and mustard cataplasms to the region of the kidneys. The effect of this treatment was a reduction of the febrile heat, relaxation of the skin, and softening of the pulse. The urine was reduced a quart a day from the former quantity, and the coagulable matter disappeared. It became, however, more sensibly saccharine. The phosphate of iron was now administered, in combination with a light bitter and rhubarb. At first it was given in doses of three grains daily, afterwards fifteen grains. The improvement soon became remarkable—the febrile thirst and the keen appetite disappeared—the urine greatly diminished, and void of its saccharine quality. The patient soon regained flesh and strength, and quite recovered.

*Case* 5. A. Sandys, aged eight years, complains of weakness, head-ache, cough, dyspnœa, and a variety of other symptoms simulating phthisical affection. There was great emaciation, urgent thirst, voracious appetite, fever, hard pulse, furred tongue, and constipated bowels. The alvine discharges were unnatural, being almost as dark as pitch—abdomen full and prominent—pain in the right hypochondrium, with manifest fulness there. The urine measured from six to eight pints in the 24 hours, being sweetish, pale, and transparent. There was pain in the loins and aching of the spine, when in motion. Pressure on the region of the kidneys excited nausea and even vomiting. This child had been delicate from birth. She had lately had small-pox, hooping-cough, scarlatina, and measles, after which the pulmonary symptoms had come on. Blood was taken from the arm, and leeches applied to the hypochondrium. Small doses of calomel were also prescribed to correct the state of the liver and bowels; but it produced irritation and was left off. All other preparation of mercury also disagreed. The taraxacum and chlorine succeeded better, and the fæces became natural. No alteration in the urinary secretion—the diuresis continued excessive. The phosphate of iron, in doses of three grains, thrice a day, was given, and leeches were applied to the loins. The phosphate was gradually increased. But although it controlled the urinary discharge, it oppressed the stomach, which effect was relieved by a mustard cataplasm to that region. The sweetness of the urine was gradually but slowly removed under the continued use of the phosphate, and the discharge itself considerably reduced. Amendment took place to a certain point, but could not be pushed further. An issue was therefore inserted on each side of the spine. The general health was supported by bark, and the phosphate was continued. At the end of five months the little patient was so far convalescent that the caustic issues were removed—she was sent to the sea, whence she returned perfectly well.

We have now furnished our readers with a few examples of the numerous cases detailed at length by Dr. Venables, and also an outline of the principles and modes of treatment which he has laid down. The young practitioner in particular will do well to peruse the whole work, and indeed much information will be derived from it by every class of the profession. We tender the able author our thanks for the pleasure and instruction we have received from an examination of the publication, and beg to express to him our sincere wishes for a continuance of that zeal and ability which he has shewn in the prosecution of medical science.

